# Prognostic performance of computerized tomography scoring systems in civilian penetrating traumatic brain injury: an observational study

**DOI:** 10.1007/s00701-019-04074-1

**Published:** 2019-10-28

**Authors:** Matias Lindfors, Caroline Lindblad, David W. Nelson, Bo-Michael Bellander, Jari Siironen, Rahul Raj, Eric P. Thelin

**Affiliations:** 1 Department of Neurosurgery, Helsinki University Hospital and University of Helsinki, Topeliuksenkatu 5, PB 266, 00029 Helsinki, Finland; 2 Department of Clinical Neuroscience, Karolinska Institutet, Stockholm, Sweden; 3 Department of Physiology and Pharmacology, Section of Perioperative Medicine and Intensive Care, Karolinska Institutet, Stockholm, Sweden; 4 Function Perioperative Medicine and Intensive Care, Karolinska University Hospital, Stockholm, Sweden; 5 Department of Neurosurgery, Karolinska University Hospital, Stockholm, Sweden; 6 Division of Neurosurgery, Department of Clinical Neurosciences, University of Cambridge, Cambridge Biomedical Campus, Cambridge, UK; 7 Theme Neuro, Karolinska University Hospital, Stockholm, Sweden

**Keywords:** Traumatic brain injury, Penetrating traumatic brain injury, Computerized tomography, Prognosis, Outcome prediction

## Abstract

**Background:**

The prognosis of penetrating traumatic brain injury (pTBI) is poor yet highly variable. Current computerized tomography (CT) severity scores are commonly not used for pTBI prognostication but may provide important clinical information in these cohorts.

**Methods:**

All consecutive pTBI patients from two large neurotrauma databases (Helsinki 1999–2015, Stockholm 2005–2014) were included. Outcome measures were 6-month mortality and unfavorable outcome (Glasgow Outcome Scale 1–3). Admission head CT scans were assessed according to the following: Marshall CT classification, Rotterdam CT score, Stockholm CT score, and Helsinki CT score. The discrimination (area under the receiver operating curve, AUC) and explanatory variance (pseudo-*R*^2^) of the CT scores were assessed individually and in addition to a base model including age, motor response, and pupil responsiveness.

**Results:**

Altogether, 75 patients were included. Overall 6-month mortality and unfavorable outcome were 45% and 61% for all patients, and 31% and 51% for actively treated patients. The CT scores’ AUCs and pseudo-*R*^2^s varied between 0.77–0.90 and 0.35–0.60 for mortality prediction and between 0.85–0.89 and 0.50–0.57 for unfavorable outcome prediction. The base model showed excellent performance for mortality (AUC 0.94, pseudo-*R*^2^ 0.71) and unfavorable outcome (AUC 0.89, pseudo-*R*^2^ 0.53) prediction. None of the CT scores increased the base model’s AUC (*p* > 0.05) yet increased its pseudo-*R*^2^ (0.09–0.15) for unfavorable outcome prediction.

**Conclusion:**

Existing head CT scores demonstrate good-to-excellent performance in 6-month outcome prediction in pTBI patients. However, they do not add independent information to known outcome predictors, indicating that a unique score capturing the intracranial severity in pTBI may be warranted.

**Electronic supplementary material:**

The online version of this article (10.1007/s00701-019-04074-1) contains supplementary material, which is available to authorized users.

## Introduction

Traumatic brain injury (TBI) constitutes a leading cause of death and long-term disability worldwide [24, 25]. Although the majority of TBIs are blunt, civilian penetrating injuries are increasing, especially in the USA [24], and represent a considerable proportion of TBI mortality and all trauma-related deaths [4, 20]. Compared with blunt TBIs, penetrating TBIs (pTBI) are associated with significantly higher rates of morbidity and mortality. Up to 71–90% of patients die either at the scene of accident or during transportation [1, 12, 38, 41] and reported inpatient mortality rates range from 22 to 84% [1, 8, 9, 11, 16, 18, 19, 28, 30, 31, 35, 38–42, 45].

Given the poor yet variable outcomes accompanying pTBI, accurate prognostication is crucial in determining which patients are likely to benefit from aggressive therapeutic interventions. However, studies into prognostic assessments in pTBI are scarce and not as thorough as studies on blunt TBI [32, 34, 43]. Instead, they are often based on small or relatively outdated single-center series [2, 3, 9, 10, 12, 18, 19, 28, 39–41], save some exceptions [1, 11, 26, 42]. Moreover, to the best of our knowledge, the performance of previously developed head computerized tomography (CT) classification schemes in outcome prediction has not been assessed outside blunt TBI cohorts [33, 36, 44].

The primary aim of this study was to assess the prognostic performance of previously developed head CT scoring systems in a contemporary two-center cohort of patients with civilian pTBI admitted to academic neurosurgical intensive care units (ICU). We specifically aimed to evaluate the performance of four head CT classification systems (Marshall CT classification [27], Rotterdam CT score [23], Stockholm CT score [33], Helsinki CT score [36]) in predicting 6-month mortality and 6-month functional outcome independently and together with known TBI outcome predictors.

## Materials and methods

### Study design and setting

This retrospective observational two-center study investigated the prognostic performance of specific head CT scoring systems in civilian pTBI. Both participating centers (Töölö Hospital of HUS-Helsinki University Hospital [HUS], Helsinki, Finland; Karolinska University Hospital [KUH], Stockholm, Sweden) are the only tertiary trauma centers providing specialist neurosurgical and neurointensive care in their respective regions, encompassing a combined catchment area population of nearly 4 million inhabitants. The healthcare systems of both countries are publicly funded, and the hospitals are non-profit in nature, providing treatment to all citizens regardless of socioeconomic factors or insurance status. The treatment of pTBI in both centers adheres to treatment guidelines resembling those that have recently been published [17].

### Study population and data collection

**Fig. 1:**
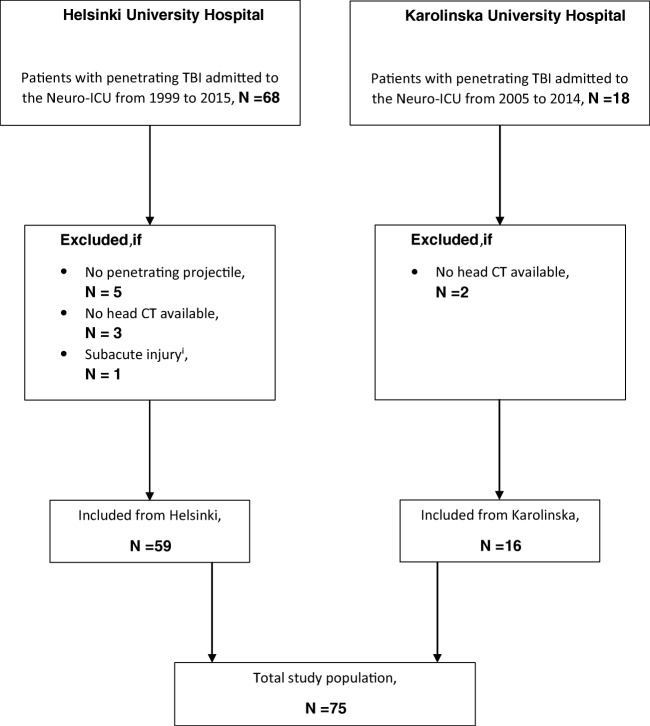
Flowchart demonstrating the inclusion and exclusion of patients. CT, computerized tomography; neuro-ICU, neurosurgical intensive care unit; TBI, traumatic brain injury

All patients with pTBI admitted to the neurosurgical ICU of either HUS between 1 January 1999 and 31 December 2015 or KUH between 1 January 2005 and 31 December 2014 were included in this study. Patients were identified from databases that have been previously described [22, 44]. A pTBI was defined as an injury in which a projectile penetrates the skull and enters the intracranial space. All patients’ admission head CT scans were reviewed to verify the diagnosis. Patients who died prior to ICU admission and patients who were readmitted or primarily treated at another neurosurgical center were not considered. We further excluded patients presenting more than 24 h after injury, and patients whose admission head CT scans were either missing or demonstrated no intracranial penetration (Fig. 1) (**SDC 1**).

Patient-level data were obtained from existing TBI databases, including data on patient demographics, type of weapon, and inflictor of injury. Both databases contain admission characteristics according to the International Mission for Prognosis and Analysis of Clinical Trials in TBI (IMPACT) prognostic models [13].

Admission head CT scans were reviewed by a set of pre-defined characteristics depicting projectile trajectory and enabling the computation of all four CT scores under investigation. Furthermore, each patient’s angiographic studies were evaluated for arterial injuries when available. Two authors (ML and RR) assessed all imaging studies in the HUS cohort (Cohen’s *κ* = 0.92 [95% CI, 0.90–0.95]), and two authors (CL and EPT) assessed all imaging studies in the KUH cohort (Cohen’s *κ* = 0.90 [95% CI, 0.89–0.94]). Uncertain cases were discussed between the authors to reach a final classification/score.

At HUS, patients with pTBI triaged as moribund on arrival are routinely admitted to the neurosurgical ICU for monitoring and potential organ procurement for transplantation, even when not receiving active neurointensive care. Therefore, patients in the HUS cohort who were assigned to a standard treatment regimen were categorized as actively treated, and patients admitted as unsalvageable were categorized as inactively treated. At KUH, patients withheld from active treatment are not admitted to the ICU, and hence all patients in the KUH cohort were actively treated and categorized accordingly.

### Outcome variables

Primary outcome measures were 6-month all-cause mortality and 6-month functional outcome, assessed using the Glasgow Outcome Scale (GOS) [14]. We further report 30-day all-cause mortality. Dates of death were extracted from the Population Register Centre of Finland and the Swedish Tax Agency, both keeping records of the dates and causes of death of all Finnish and Swedish citizens, respectively. At HUS, GOS assessments were conducted at outpatient follow-up appointments, and at KUH, GOS was obtained by using a structured GOS assessment questionnaire or at follow-up appointments. GOS was dichotomized into favorable outcome (GOS 4–5) and unfavorable outcome (GOS 1–3) in the statistical analyses.

### Statistical analysis

General characteristics of the study sample are presented as medians and interquartile ranges (IQRs) for continuous variables and as numbers and percentages for categorical variables. Inter-group comparisons were conducted using Fisher’s exact test (two-tailed) when analyzing categorical data. Continuous data were tested for skewness; all data were highly skewed and hence analyzed using either the Mann–Whitney *U* test or the Kruskal–Wallis test. To counteract the increased risk of type I error associated with multiple comparisons, a Bonferroni correction was used when appropriate.

The prognostic performance of different head CT classification systems was assessed by determining their discrimination (using the area under the receiver operating characteristic curve [AUC]) and explanatory variance (using the Nagelkerke’s pseudo-*R*^2^, referred to as “pseudo-*R*^2^”).

Each CT classification system was assessed for both univariate performance and independent prognostic performance in reference to an established base model consisting of age (continuous variable), GCS motor score (continuous variable), and pupil responsiveness [43]. The Marshall CT classification and Rotterdam CT score were analyzed as categorical variables, the Rotterdam CT score being ordinal, and the Helsinki CT score and Stockholm CT score were analyzed as continuous variables, as has been previously suggested [44]. Differences in AUC were compared using the DeLong test [7].

All analyses were performed using SPSS Statistics for Windows, version 24.0, released 2017 (IBM Corp, Armonk, NY, USA), or RStudio® (R Foundation for Statistical Computing, Vienna, Austria; https://www.r-project.org/). Missing data were excluded from all analyses; no imputations were conducted due to the small sample size. A two-tailed *p* value of ≤ 0.05 was considered statistically significant.

### Ethical considerations

The regional ethics committees in both Helsinki (123/13/03/02/2016 TMK02 § 80) and Stockholm (2016/999-31/4), (2018/2074-32) approved the study and waived the need for informed consent. The study adheres to the STrengthening the Reporting of OBservational studies in Epidemiology (STROBE) statement (**SDC 2**).

## Results

### Study population characteristics

A total of 75 patients were included. A detailed description of study sample characteristics is presented in Table 1. Admission and head CT characteristics were similar between the two study centers. Patient median age was 41 years and 91% of patients were male. Altogether, 64% of injuries were self-inflicted and 68% of patients had firearm-related injuries. In total, 53% of patients presented with a GCS score of 3–8, while 32% of patients had an admission GCS score of 13–15 and 49% had normal pupil responsiveness. Notably, all elderly patients (> 60 years) were male and had self-inflicted firearm-related injuries (**SDC 3**). Moreover, patients with self-inflicted injuries were significantly older than patients with non-self-inflicted injury (median age 47 versus 26 years, *p* < 0.001) (**SDC 4**).

**Table 1:** Patients baseline characteristics

Parameter	Combined cohort (*N* = 75)	Helsinki cohort (*N* = 59)	Stockholm cohort (*N* = 16)	*p* value	Active treatment cohort (*N* = 59)	Inactive treatment cohort (*N* = 16)	*p* value
Demography							
Age	41.0 (26.0–52.0)	41.0 (26.0–51.0)	42.5 (26.0–55.0)	0.637	41.0 (26.0–53.0)	41.5 (27.0–47.0)	0.796
Sex							
Male	68 (91%)	54 (92%)	14 (88%)	0.637	53 (90%)	15 (94%)	1.000
Female	7 (9%)	5 (9%)	2 (13%)		6 (10%)	1 (6%)	
Admission							
Weapon type							
Firearm	51 (68%)	40 (68%)	11 (69%)	0.129	35 (59%)	16 (100%)	0.025
Nail gun	10 (13%)	10 (17%)	0		10 (17%)	0	
Sharp object	10 (13%)	7 (12%)	3 (19%)		10 (17%)	0	
Other	4 (5%)	2 (3%)	2 (13%)		4 (7%)	0	
Self-inflicted injury^a^	48 (64%)	41 (69%)	7 (44%)	0.214	37 (63%)	11 (69%)	1.000
Pre-hospital physician involvement^b^	51 (68%)	37 (63%)	14 (88%)	0.028	39 (66%)	12 (75%)	0.762
Inter-hospital transfer	14 (19%)	11 (19%)	3 (19%)	1.000	13 (24%)	0	0.032
Admission delay							
< 1 h	18 (24%)	16 (27%)	2 (13%)	0.181	13 (22%)	5 (31%)	0.103
1–2 h	36 (48%)	25 (42%)	11 (69%)		26 (44%)	10 (63%)	
>2 h	19 (25%)	17 (29%)	3 (19%)		18 (31%)	1 (6%)	
Missing	2 (3%)	1 (2%)	1 (6%)		2 (3%)	0	
GCS score							
3–8	40 (53%)	32 (54%)	8 (50%)	0.793	24 (41%)	16 (100%)	< 0.001
9–12	10 (13%)	7 (12%)	3 (19%)		10 (17%)	0	
13–15	24 (32%)	19 (32%)	5 (31%)		24 (41%)	0	
Missing	1 (1%)	1 (2%)	0		1 (2%)	0	
GCS motor scale							
1	20 (27%)	16 (27%)	4 (25%)	0.289	8 (14%)	12 (75%)	< 0.001
2	10 (13%)	7 (12%)	3 (19%)		6 (10%)	4 (25%)	
3	1 (1%)	0	1 (6%)		1 (2%)	0	
4	8 (11%)	8 (14%)	0		8 (14%)	0	
5	8 (11%)	7 (12%)	1 (6%)		8 (14%)	0	
6	28 (37%)	21 (36%)	7 (44%)		28 (48%)	0	
Missing	0	0	0		0	0	
Pupil responsiveness							
Both	37 (49%)	31 (53%)	6 (38%)	0.453	36 (61%)	1 (6%)	<0.001
One	8 (11%)	7 (12%)	1 (6%)		7 (12%)	1 (6%)	
None	27 (36%)	19 (32%)	8 (50%)		13 (22%)	14 (88%)	
Missing	3 (4%)	2 (3%)	1 (6%)		3 (5%)	0	
Hypotension^a, c^	17 (23%)	13 (22%)	4 (25%)	1.000	14 (24%)	3 (19%)	1.000
Hypoxia^d, e^	13 (17%)	12 (20%)	1 (6%)	0.673	7 (12%)	6 (38%)	0.065
Coagulopathy^f, g^	8 (11%)	6 (10%)	2 (13%)	0.615	7 (12%)	1 (6%)	0.673
Radiology							
Perforating	26 (35%)	22 (37%)	4 (25%)	0.555	15 (25%)	11 (69%)	0.002
Entry							
Frontobasal	26 (35%)	22 (37%)	4 (25%)	0.528	20 (34%)	6 (38%)	0.062
Temporal	35 (47%)	27 (46%)	8 (50%)		25 (42%)	10 (63%)	
Other	14 (19%)	10 (17%)	4 (25%)		14 (24%)	0	
Exit							
Frontobasal	7 (9%)	6 (10)	1 (6%)	0.755	5 (9%)	2 (13%)	0.004
Temporal	11 (15%)	10 (17%)	1 (6%)		5 (9%)	6 (38%)	
Other	8 (11%)	6 (10%)	2 (13%)		5 (9%)	3 (19%)	
Trajectory							
Monohemispheric	39 (52%)	32 (54%)	7 (44%)	0.575	35 (59%)	4 (25%)	0.023
Bihemispheric	34 (45%)	27 (46%)	7 (44%)	1.000	22 (37%)	12 (75%)	0.010
Unilobar	18 (24%)	14 (24%)	4 (25%)	1.000	18 (31%)	0	0.008
Multilobar	55 (73%)	45 (76%)	10 (63%)	0.341	39 (66%)	16 (100%)	0.004
Posterior fossa	14 (19%)	7 (12%)	7 (44%)	0.008	14 (24%)	0	0.032
Transventricular	33 (44%)	28 (48%)	5 (31%)	0.273	21 (36%)	12 (75%)	0.009
In proximity to COW^h^	25 (33%)	20 (34%)	5 (31%)	1.000	19 (32%)	6 (38%)	0.768
Bone or projectile fragments present	65 (87%)	53 (90%)	12 (75%)	0.206	49 (83%)	16 (100%)	0.081
Basal cisterns							
Normal	25 (33%)	19 (32%)	6 (38%)	0.067	25 (42%)	0	< 0.001
Compressed	36 (48%)	26 (44%)	10 (63%)		32 (54%)	4 (25%)	
Obliterated	14 (19%)	14 (24%)	0		2 (3%)	12 (75%)	
Midline shift							
0 mm	41 (53%)	33 (56%)	7 (44%)	0.753	34 (58%)	6 (38%)	0.031
1–5 mm	10 (13%)	7 (12%)	3 (19%)		9 (15%)	1 (6%)	
5–10 mm	17 (23%)	13 (22%)	4 (25%)		13 (22%)	4 (25%)	
> 10 mm	8 (11%)	6 (10%)	2 (13%)		3 (5%)	5 (31%)	
Mass lesion > 25 cm^3^	23 (31%)	18 (31%)	5 (31%)	1.000	11 (19%)	12 (75%)	< 0.001
EDH	2 (3%)	0	2 (13%)	0.043	2 (3%)	0	1.000
SDH	48 (64%)	36 (61%)	12 (75%)	0.386	32 (54%)	16 (100%)	< 0.001
ICH	56 (75%)	44 (75%)	12 (75%)	1.000	41 (70%)	15 (94%)	0.056
Bilateral SDH	10 (15%)	10 (17%)	1 (6%)	0.439	4 (7%)	7 (44%)	0.001
tSAH in convexities							
0 mm	13 (17%)	11 (19%)	2 (13%)	0.004	13 (22%)	0	0.013
1–5 mm	15 (20%)	7 (12%)	8 (50%)		14 (24%)	1 (6%)	
> 5 mm	47 (63%)	41 (70%)	6 (38%)		32 (54%)	15 (94%)	
tSAH in basal cisterns							
0 mm	41 (55%)	33 (56%)	8 (50%)	0.189	34 (58%)	7 (44%)	0.433
1–5 mm	9 (12%)	5 (9%)	4 (25%)		6 (10%)	3 (19%)	
> 5 mm	25 (33%)	21 (36%)	4 (25%)		19 (32%)	6 (38%)	
IVH	39 (52%)	33 (56%)	6 (38%)	0.261	24 (41%)	15 (94%)	< 0.001
Leroux IVH score							
0	36 (48%)	26 (44%)	10 (63%)	0.048	35 (59%)	1 (6%)	< 0.001
1–10	23 (31%)	17 (29%)	6 (38%)		18 (31%)	5 (31%)	
> 10	16 (21%)	16 (27%)	0		6 (10%)	10 (63%)	
Acute hydrocephalus	19 (25%)	9 (15%)	10 (63%)	<0.001	14 (24%)	5 (31%)	0.533
DAI	0	0	0	NA	0	0	NA
CTA performed	19 (25%)	17 (29%)	2 (13%)	0.330	16 (27%)	3 (19%)	0.747
DSA performed	10 (13%)	10 (17%)	0	0.107	10 (17%)	0	0.107
Confirmed arterial injury	6 (8%)	6 (10%)	0	0.331	5 (9%)	1 (6%)	1.000
Marshall CT classification							
I	0	0	0	0.458	0	0	< 0.001
II	22 (29%)	16 (27%)	6 (38%)		22 (37%)	0	
III	20 (27%)	18 (31%)	2 (13%)		17 (29%)	3 (19%)	
IV	10 (13%)	7 (12%)	3 (19%)		9 (15%)	1 (6%)	
V or VI	23 (31%)	18 (31%)	5 (31%)		11 (19%)	12 (75%)	
Rotterdam CT score							
1	0	0	0	0.640	0	0	< 0.001
2	9 (12%)	6 (10%)	3 (19%)		9 (15%)	0	
3	13 (17%)	10 (17%)	3 (19%)		13 (22%)	0	
4	23 (31%)	19 (32%)	4 (25%)		22 (37%)	1 (6%)	
5	24 (32%)	18 (31%)	6 (38%)		15 (25%)	9 (56%)	
6	6 (8%)	6 (10%)	0		0	6 (38%)	
Helsinki CT score	6 (3–10)	6 (3–10)	6 (3–8)	0.324	5 (2–8)	13 (10–14)	< 0.001
Stockholm CT score	3.2 (2.0–4.0)	3.2 (2.0–4.0)	3.1 (1.5–4.2)	0.315	2.6 (2.0–4.0)	4.4 (4.0–5.1)	< 0.001

Categorical data presented as *N* (%) and continuous variables presented as median (IRQ). *COW*, circle of Willis; *CT*, computerized tomography; *CTA*, computerized tomography angiography; *DAI*, diffuse axonal injury; *DSA*, digital subtraction angiography; *EDH*, epidural hematoma; *GCS*, Glasgow Coma Scale; *ICH*, intracerebral hematoma; *IVH*, intraventricular hemorrhage; *SDH*, subdural hematoma; *tSAH*, traumatic subarachnoid hemorrhage

Data missing for ^a^ = 2, ^b^ = 1, ^d^ = 8, ^f^ = 4 patients

^c^Systolic blood pressure < 90 mmHg at any time prior to admission

^e^Blood oxygen saturation < 90% at any time prior to admission

^g^International normalized ratio ≥ 1.5 or activated partial thromboplastin time > 36 s or thrombocyte count < 100,000 mm^3^

^h^Within 2 cm of COW

Overall, 79% of patients were actively treated. All patients from whom active treatment was withheld had firearm-related injuries, a GCS motor score of 1 or 2, and 88% had no pupil responsiveness (Table 1). In patients who were actively treated, 76% underwent a debridement operation and 7% underwent a decompressive craniectomy (**SDC 5)**. Median ICU length of stay was 5 days (IQR 1–10) and median hospital length of stay was 8 days (IQR 5–17) for those who received active treatment.

Radiologically, the wound trajectory was perforating (i.e., including an entry and an exit wound) in 35% of patients, bihemispheric in 45% of patients, and transventricular in 44% of patients, all of which were significantly more common in patients with a GCS score of 3–8 (**SDC 6**). Frontobasal and temporal entry regions accounted for 35% and 47% of all injuries, respectively, with frontobasal entry sites being more common in patients with self-inflicted injuries (**SDC 4**). Moreover, patients with injuries resulting from firearms or sharp objects had higher intracranial injury severity than those with other modes of injury, irrespective of the CT classification scheme applied (**SDC 7**).

### Outcomes

**Fig. 2:**
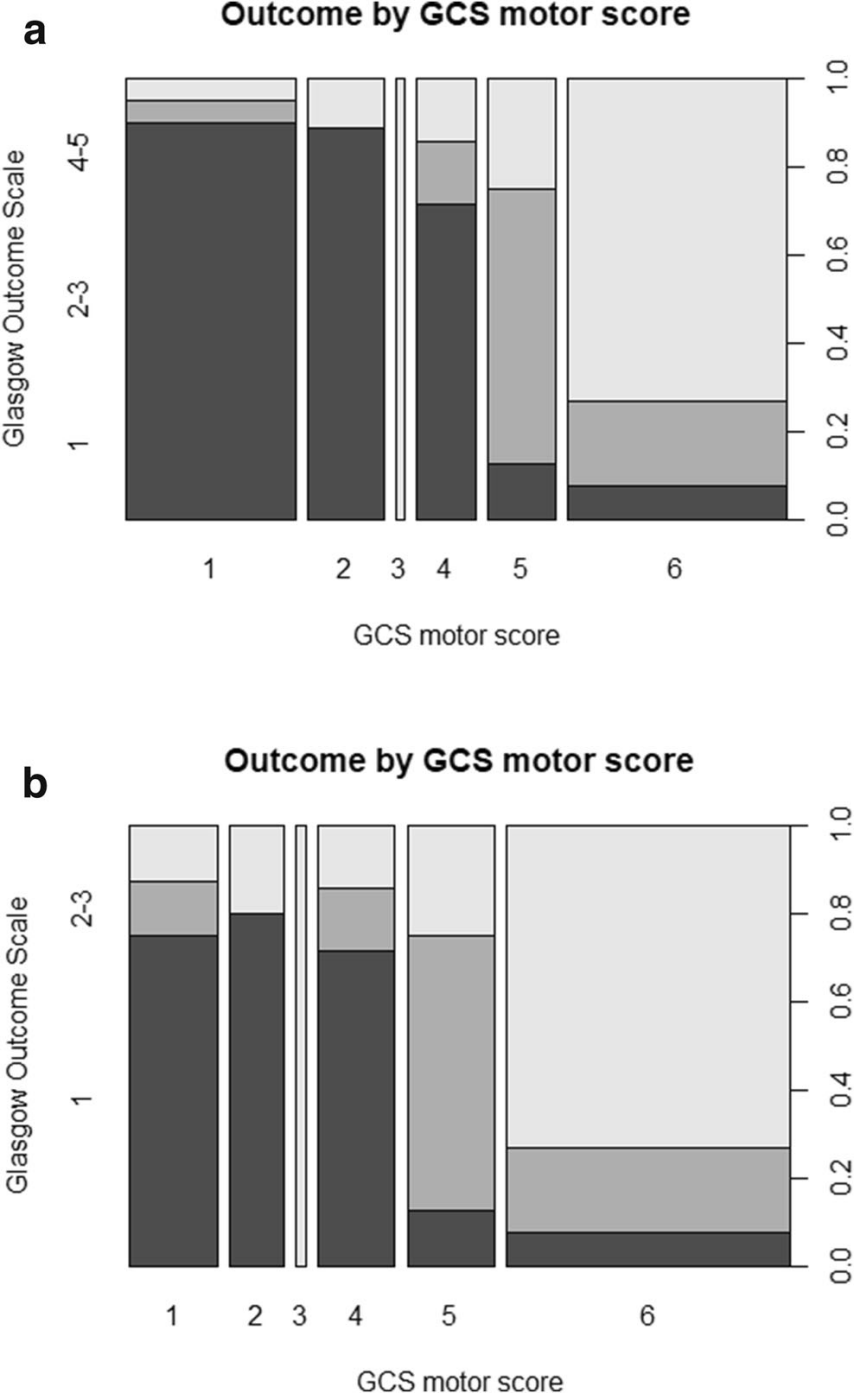
Spine plots illustrating the relationship between GCS motor score (*x*-axis) and functional outcome (*y*-axis, left) for the complete cohort (**a**) and the active treatment cohort (**b**). The right *y*-axis represents outcome proportions summing to 1. On the left *y*-axis, dark gray represents a GOS of 1, medium gray represents a GOS of 2 or 3, and light gray represents a GOS of 4 or 5. The sizes of the bins correspond to the number of patients in each category. GCS, Glasgow Coma Scale; GOS, Glasgow Outcome Scale

**Fig. 3:**
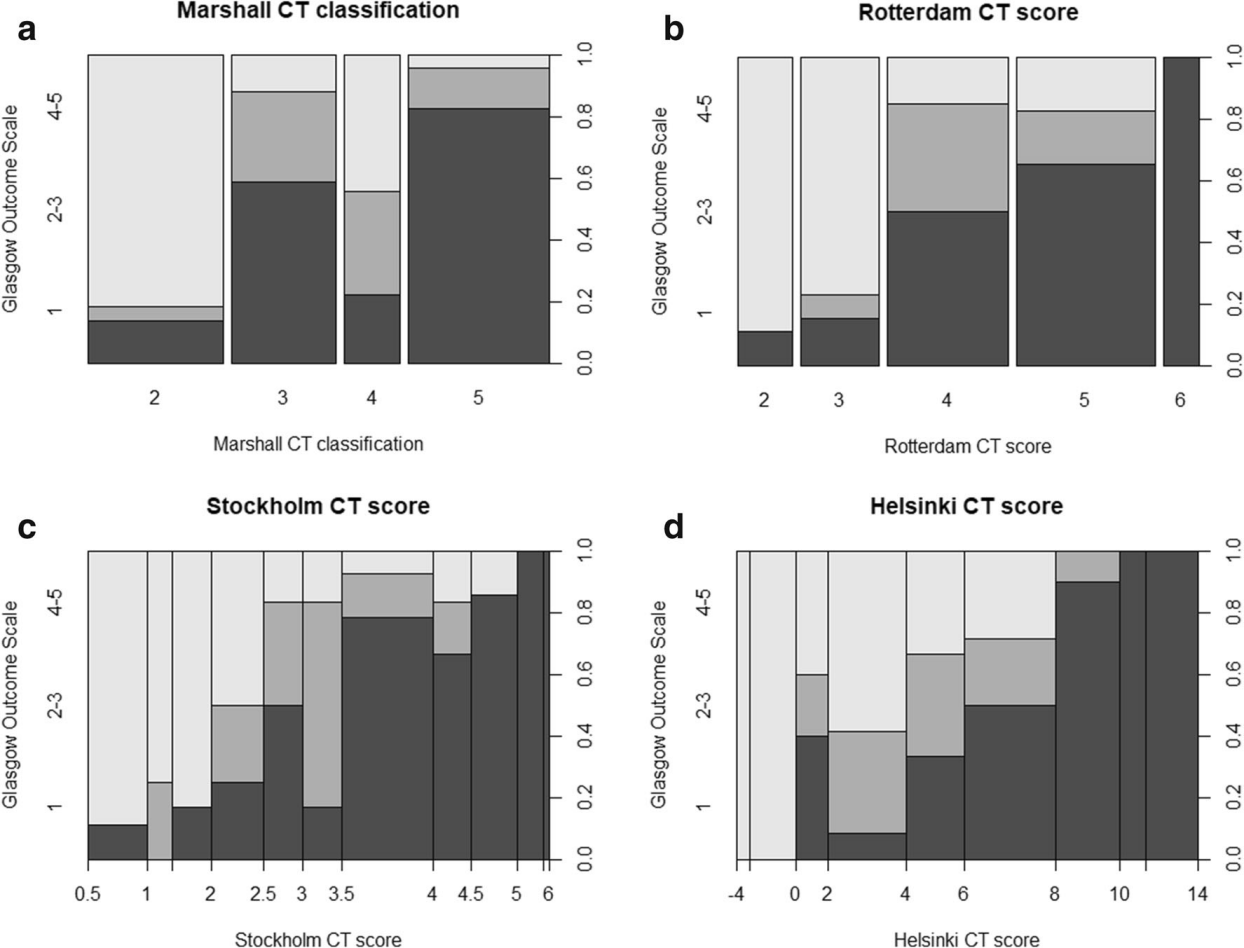
Spine plots illustrating the relationship between CT findings (*x*-axis) and functional outcome (*y*-axis, left) for the Marshall CT classification (**a**), the Rotterdam CT score (**b**), the Stockholm CT score (**c**), and the Helsinki CT score (**d**). The right *y*-axis represents outcome proportions summing to 1. On the left *y*-axis, dark gray represents a GOS of 1, medium gray represents a GOS of 2 or 3, and light gray represents a GOS of 4 or 5. The sizes of the bins correspond to the number of patients in each category. CT, computerized tomography; GOS, Glasgow Outcome Scale

In the complete cohort, unadjusted 6-month all-cause mortality was 45% and total unfavorable outcome was 61%. In the active treatment cohort, 6-month mortality was 31% and total unfavorable outcome was 51% (Table 2). There was no difference between 30-day and 6-month mortality; all deaths occurred within the first month after injury. Higher rates of both mortality and unfavorable outcome were observed in elderly patients and in patients with either self-inflicted or firearm-related injuries, low GCS motor scores (Fig. 2), or high intracranial injury severity (Fig. 3). By contrast, out of patients with mild injury (GCS 13–15), only one patient (4%) died and only five patients (21%) were dependent (GOS 3) at 6 months post-injury.

**Table 2:** Patient outcomes

	Complete cohort (*N* = 75)		Active treatment cohort (*N* = 59)	
	6-month all-cause mortality^a^	6-month unfavorable outcome*	6-month all-cause mortality^a^	6-month unfavorable outcome*
Overall	34 (45%)	46 (61%)	18 (31%)	30 (51%)
Center subgroups				
Helsinki	27 (46%)	35 (59%)	11 (26%)	19 (44%)
Stockholm	7 (44%)	11 (69%)	7 (44%)	11 (69%)
Age subgroups				
≤ 40 years	14 (39%)	17 (47%)	7 (24%)	10 (35%)
41–60 years	9 (32%)	18 (64%)	3 (14%)	12 (55%)
> 60 years	11 (100%)	11 (100%)	8 (100%)	8 (100%)
Weapon subgroups				
Firearm	31 (61%)	38 (75%)	15 (43%)	22 (63%)
Nail gun	1 (10%)	2 (20%)	1 (10%)	2 (20%)
Sharp object	2 (20%)	5 (50%)	2 (20%)	5 (50%)
Other	0	1 (25%)	0	1 (25%)
Self-inflicted subgroups				
Yes	25 (52%)	32 (67%)	14 (38%)	21 (57%)
No	8 (32%)	12 (48%)	3 (15%)	7 (35%)
GCS subgroups				
3–8	31 (78%)	34 (85%)	15 (63%)	18 (75%)
9–12	2 (20%)	6 (60%)	2 (20%)	6 (60%)
13–15	1 (4%)	5 (21%)	1 (4%)	5 (21%)

*GCS*, Glasgow Coma Scale; *GOS*, Glasgow Outcome Scale

^a^Identical to 30-day all-cause mortality

*Defined as GOS 1–3; missing for 4 patients; median time to follow-up for 6-month survivors was 302 days (IQR 188–388 days)

### Prognostic performance of CT classification systems

Discrimination and overall performance measures of univariate models are presented in Table 3. Generally, all CT scoring systems demonstrated better performance in the complete cohort in comparison with active treatment cohort, irrespective of the outcome dichotomization.

**Table 3:** Univariate performance of CT models

Model	Complete cohort			Active treatment cohort		
	*R*^2^	AUC (95% CI)	*p* value	*R*^2^	AUC (95% CI)	*p* value
6-month mortality						
Marshall	0.402	0.815 (0.715–0.914)	0.046	0.247	0.750 (0.612–0.888)	0.362
Rotterdam	0.348	0.774 (0.669–0.879)	0.003	0.119	0.654 (0.509–0.799)	0.037
Stockholm	0.459	0.850 (0.827–0.973)	0.089	0.287	0.783 (0.653–0.912)	0.390
Helsinki	0.601	0.900 (0.762–0.938)	Ref	0.368	0.816 (0.694–0.939)	Ref
6-month unfavorable outcome*						
Marshall	0.574	0.887 (0.802–0.971)	Ref	0.498	0.849 (0.742–0.957)	Ref
Rotterdam	0.519	0.846 (0.744–0.947)	0.116	0.443	0.825 (0.710–0.941)	0.366
Stockholm	0.507	0.871 (0.776–0.967)	0.769	0.407	0.833 (0.718–0.949)	0.802
Helsinki	0.502	0.868 (0.787–0.949)	0.653	0.363	0.800 (0.685–0.915)	0.391

Differences in AUC were compared using the DeLong test. *AUC*, area under the curve; *CI*, confidence interval; *GOS*, Glasgow Outcome Scale

*Defined as GOS 1–3; missing for 4 patients

For 6-month mortality prediction, the Helsinki CT score outperformed the three other models, exhibiting an AUC of 0.90 and a pseudo-*R*^2^ of 0.60. The differences in AUC between Helsinki CT and the other scores were statistically significant for the Marshall CT classification (*p* = 0.046) and Rotterdam CT score (*p* = 0.003), but not for the Stockholm CT score (*p* = 0.089).

For unfavorable outcome prediction, the Marshall CT classification reached an AUC of 0.89 and a pseudo-*R*^2^ of 0.57, thus performing marginally better than the Stockholm, Helsinki, and Rotterdam CT scores. However, the differences in AUC between the CT scores were not statistically significant (*p* > 0.05 for all).

The base model consisting of age, GCS motor score, and pupil responsiveness demonstrated an AUC of 0.94 and a pseudo-*R*^2^ of 0.71 for 6-month mortality prediction, and an AUC of 0.89 and a pseudo-*R*^2^ of 0.53 for unfavorable outcome prediction (Table 4). None of the CT classification schemes provided a significant increase in AUC to the base model for mortality or unfavorable outcome prediction (*p* > 0.05 for all). Still, concerning unfavorable outcome prediction, the addition of all CT models slightly increased the base model’s pseudo-*R*^2^ (+ 0.09–0.15 for the complete cohort and + 0.11–0.19 for the active treatment cohort).

**Table 4:** Multivariate performance of CT models

Model	Complete cohort					Active treatment cohort				
	*R*^2^	Gain in *R*^2^	AUC (95% CI)	Gain in AUC	*p* value	*R*^2^	Gain in *R*^2^	AUC (95% CI)	Gain in AUC	*p* value
6-month mortality										
Base	0.708		0.943 (0.896–0.991)			0.578		0.917 (0.847–0.987)		
Base + Marshall	0.739	+ 0.031	0.947 (0.902–0.992)	+ 0.004	0.720	0.608	+ 0.030	0.911 (0.837–0.985)	− 0.006	0.749
Base + Rotterdam	0.753	+ 0.045	0.953 (0.911–0.995)	+ 0.010	0.328	0.588	+ 0.010	0.914 (0.842–0.986)	− 0.003	0.719
Base + Helsinki	0.792	+ 0.084	0.963 (0.928–0.999)	+ 0.020	0.220	0.668	+ 0.090	0.931 (0.868–0.993)	+ 0.014	0.588
Base + Stockholm	0.741	+ 0.033	0.952 (0.909–0.994)	+ 0.009	0.410	0.611	+ 0.033	0.919 (0.849–0.988)	+ 0.002	0.933
6-month unfavorable outcome*										
Base	0.526		0.885 (0.806–0.964)			0.405		0.823 (0.709–0.937)		
Base + Marshall	0.673	+ 0.147	0.933 (0.876–0.990)	+ 0.048	0.099	0.594	+ 0.189	0.898 (0.813–0.983)	+ 0.075	0.093
Base + Rotterdam	0.672	+ 0.146	0.930 (0.869–0.992)	+ 0.045	0.124	0.590	+ 0.185	0.892 (0.802–0.982)	+ 0.069	0.123
Base + Helsinki	0.619	+ 0.093	0.917 (0.846–0.988)	+ 0.032	0.159	0.514	+ 0.109	0.876 (0.771–0.980)	+ 0.053	0.121
Base + Stockholm	0.639	+ 0.112	0.927 (0.857–0.996)	+ 0.042	0.130	0.545	+ 0.140	0.901 (0.808–0.994)	+ 0.078	0.052

Differences in AUC were compared using the DeLong test. Base model: age + GCS motor score + pupil responsiveness. *AUC*, area under the curve; *CI*, confidence interval; *CT*, computerized tomography; *GOS*, Glasgow Outcome Scale

*Defined as GOS 1–3; missing for 4 patients

## Discussion

In this study, we assessed the prognostic performance of four head CT scoring systems in a contemporary two-center cohort of ICU-treated patients with civilian pTBI. In terms of outcome, we observed a 6-month mortality rate of 31% and an overall 6-month unfavorable outcome rate of 51%, in patients who were actively treated. Notably, all deaths occurred within 30 days from sustaining the injury. We found that all CT classification systems demonstrated good performance in predicting 6-month unfavorable outcome, with no significant difference between the individual CT scores. By contrast, for 6-month mortality prediction, the Helsinki CT score showed slightly better performance than the other CT scores. However, none of the tested CT scoring systems significantly increased the discriminatory performance of the reference model for 6-month mortality or unfavorable outcome prediction, highlighting the importance of clinical characteristics in prognosis evaluation of pTBI patients, and the possible utility of a more tailored CT scoring system for pTBI.

Previous studies into outcomes following civilian pTBI have demonstrated marked variation in both the scope of included patients and, consequently, in rates of mortality and unfavorable outcome. Generally, unselected series including patients dying at the scene of accident or during transportation report overall mortality rates between 91 and 97% [1, 3, 12, 41], whereas in neurosurgical cohorts, mortality ranges from 34 to 84% [1, 8, 9, 11, 16, 18, 28, 30, 31, 35, 39–42] and unfavorable outcome from 58 to 87% [11, 12, 28, 35, 39]. In our study, we observed a 6-month mortality rate of 31% and an overall 6-month unfavorable outcome rate of 51%, both among the lowest figures published to date, although 6-month mortality increased to 45% and unfavorable outcome to 61% when including patients who were not actively treated. These low figures are most likely explained by the fact that we only included patients admitted to the ICU, as prior studies have suggested 53–77% of patients with pTBI to die before ICU admission [10, 40]. Also, studies excluding patients dying before a head CT scan or patients considered near death have yielded results comparable with ours, with mortality rates between 35 and 43% [18, 21]. Moreover, our study included a relatively low proportion of patients with firearm-related injuries and a rather high proportion of patients with an admission GCS score of 13–15. It is well established that gunshot injuries carry an especially poor prognosis, a consequence of high projectile energy and, as a result, a greater degree of tissue destruction [46], while patients with injuries caused by low-velocity projectiles and patients with high admission GCS scores have been reported to exhibit mortality and unfavorable outcome rates as low as 18% [5]. Thus, it appears that with current treatment selection criteria, conscious patients (GCS score > 8) with pTBI who reach active neurosurgical and ICU care face a prognosis comparable with that of patients with non-penetrating TBI [37].

To date, no studies have evaluated the prognostic performance of existing head CT scoring systems in predicting outcomes following pTBI. Several studies have, however, assessed the scores’ performance in cohorts of non-penetrating TBI patients, reporting AUCs ranging primarily from 0.60 to 0.80 for both mortality and unfavorable outcome prediction [6, 36, 44, 47]. For instance, Thelin and colleagues found the Stockholm and Helsinki CT scores superior to the more conventional Rotterdam and Marshall grading systems (AUCs, 0.72–0.77 versus 0.58–0.68; pseudo-*R*^2^s, 0.19–0.28 versus 0.03–0.15) [44] in 1115 ICU-admitted patients with blunt TBI, while one study noted an AUC of 0.85 for both the Marshall CT classification and Rotterdam CT score in predicting in-hospital mortality [29]. However, interestingly, all CT scores reached higher AUCs (0.77–0.90) and pseudo-*R*^2^s (0.35–0.60) in the present study than in the blunt TBI cohorts of prior studies, despite the scores having been originally developed for blunt TBI assessment. Although no immediate explanation for this is available, it is possible that, in penetrating injuries, intracranial destruction is more extensive, and thus a prognostic system based on head CT features is more feasible and better tiered than in blunt TBI where multiple injury characteristics are not as common. Moreover, the outcome distribution in pTBI differs markedly from that of blunt TBI—a higher proportion of patients die and less recover to an unfavorable state [35]—which may, to some extent, explain especially the Helsinki CT score's’ performance (AUC 0.90) in mortality prediction.

Altogether, prognostic models specific for pTBI are scarce. The only existing study found a base model of GCS motor score and pupil responsiveness alone to reach an AUC of 0.93 [30], a finding consistent with our results. Moreover, the same study presented a multivariable model with extremely high discriminatory performance (AUC 0.97) without including any head CT variables, suggesting accurate estimates may be attainable without radiological information. Thus, together with results from previous investigations, the present study underscores the prognostic utility of clinical characteristics in the setting of pTBI. Still, future studies should further explore the role of head CT data in prognosis evaluation and seek to combine radiological information with clinical and laboratory data, enabling the development of refined prognostic models specific to pTBI.

### Strengths and limitations

We included all consecutive ICU-admitted patients with pTBI from two large academic trauma centers, responsible for providing tertiary-level care to a combined catchment area population of approximately four million inhabitants. Thus, despite its small sample size, we consider our study to be largely representative of patients with pTBI necessitating neurosurgical and neurointensive care in Nordic countries. Moreover, our study did not limit its scope to, for instance, firearm-related or self-inflicted injuries, but instead included all modes of injury currently encountered at contemporary neurosurgical institutions. Furthermore, in addition to mortality assessment, we also evaluated functional outcome, an aspect of recovery that has been overlooked by most previous studies into pTBI.

Still, certain limitations require acknowledgement. First, we only included patients admitted to a neurosurgical ICU, due to which our results are not generalizable to the majority of patients with pTBI, most of whom die prior to ICU admission [1, 12, 38, 41]. Second, the study’s retrospective design resulted in missing data and compelled us to assess functional outcome using GOS as opposed the more refined GOS-extended [15]. Still, considering that the amount of missing data was low and that most previous studies have neglected the assessment of functional outcome altogether, these shortcomings can presumably be considered as minor. Third, although this study includes two of Northern Europe’s largest hospitals, the study population is still rather small, highlighting the rarity of pTBI in the Nordics.

## Conclusion

Selected patients with pTBI receiving active ICU treatment face a reasonable prognosis, comparable with that of patients with non-penetrating TBI. Existing head CT classification systems demonstrate mostly good-to-excellent statistical performance in outcome prediction, yet do not significantly improve the performance of a simple model based on age, motor response, and pupil responsiveness. Further prospective multicenter studies into outcomes and prognostic models for pTBI are warranted.

## Electronic supplementary material

ESM 1 Image 1. Admission head CT scan of a 25-year-old male presenting with a self-inflicted low-caliber firearm-related injury. The patient was excluded from the study as the projectile had lodged into his right optic canal and did not enter intracranial space. Admission GCS score was 14, but the patient’s right eye had no vision or pupil responsiveness due to optic nerve injury. *Abbreviations*: CT, Computerized Tomography; GCS, Glasgow Coma Scale4 (JPG 19 kb)

ESM 2 (JPG 26 kb)

ESM 3 STROBE checklist (DOC 104 kb)

ESM 4 Patient baseline characteristics by age (DOCX 37 kb)

ESM 5 Patient baseline characteristics by self-infliction (DOCX 34 kb)

ESM 6 Treatment characteristics (DOCX 23 kb)

ESM 7 Patient baseline characteristics by GCS (DOCX 35 kb)

ESM 8 Patient baseline characteristics by weapon (DOCX 38 kb)
