# Supplementary figures and images for: Prognostic performance of computerized tomography scoring systems in civilian penetrating traumatic brain injury: an observational study
Source: Acta Neurochir (Wien). 2019 Oct 28;161(12):2467–78. doi: 10.1007/s00701-019-04074-1 (PMC6874621; doi:10.1007/s00701-019-04074-1)

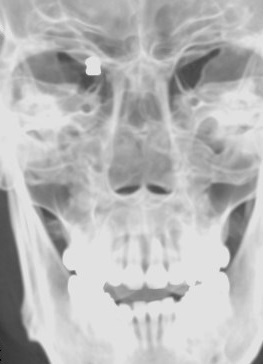

Supplement: Supplementary file 1 — Image 1. Admission head CT scan of a 25-year-old male presenting with a self-inflicted low-caliber firearm-related injury. The patient was excluded from the study as the projectile had lodged into his right optic canal and did not enter intracranial space. Admission GCS score was 14, but the patient’s right eye had no vision or pupil responsiveness due to optic nerve injury. Abbreviations: CT, Computerized Tomography; GCS, Glasgow Coma Scale4 (JPG 19 kb) [file 701_2019_4074_MOESM1_ESM.jpg]

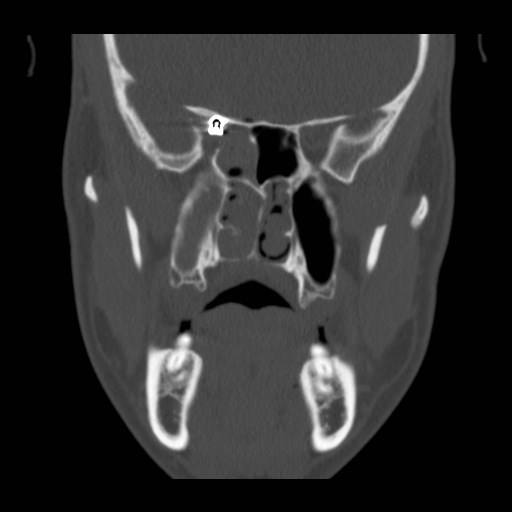

Supplement: Supplementary file 2 — (JPG 26 kb) [file 701_2019_4074_MOESM2_ESM.jpg]
