# Supplementary material for: Prognostic performance of computerized tomography scoring systems in civilian penetrating traumatic brain injury: an observational study
Source: Acta Neurochir (Wien). 2019 Oct 28;161(12):2467–78. doi: 10.1007/s00701-019-04074-1 (PMC6874621; doi:10.1007/s00701-019-04074-1)
Supplement: Supplementary file 4 — Patient baseline characteristics by age (DOCX 37 kb) [file 701_2019_4074_MOESM4_ESM.docx]

| Parameter | | Age ≤40 years (N=36) | Age 41-60 years (N=28) | Age >60 years (N=11) | *p* value |
| --- | --- | --- | --- | --- | --- |
| **Demography** | |  |  |  |  |
| Sex | |  |  |  |  |
|  | Male | 32 (89%) | 25 (89%) | 11 (100%) | 0.747 |
|  | Female | 4 (11%) | 3 (11%) | 0 |  |
| **Admission** | |  |  |  |  |
| Weapon type | |  |  |  |  |
|  | Firearm | 20 (56%) | 20 (71%) | 11 (100%) | 0.159 |
|  | Nail gun | 5 (14%) | 5 (18%) | 0 |  |
|  | Sharp object | 8 (22%) | 2 (7%) | 0 |  |
|  | Other | 3 (8%) | 1 (4%) | 0 |  |
| Self-inflicted injury^a^ | | 16 (44%) | 21 (75%) | 11 (100%) | 0.001 |
| Pre-hospital physician involvement^b^ | | 27 (75%) | 18 (64%) | 6 (55%) | 0.283 |
| Inter-hospital transfer | | 7 (19%) | 5 (18%) | 2 (18%) | 1.000 |
| Admission delay | |  |  |  |  |
|  | <1 hour | 9 (25%) | 7 (25%) | 2 (18%) | 0.788 |
|  | 1-2 hours | 19 (53%) | 13 (46%) | 4 (36%) |  |
|  | >2 hours | 7 (19%) | 8 (29%) | 4 (36%) |  |
|  | Missing | 1 (3%) | 0 | 1 (9%) |  |
| GCS score | |  |  |  |  |
|  | 3-8 | 17 (47%) | 15 (54%) | 8 (73%) | 0.074 |
|  | 9-12 | 2 (6%) | 6 (21%) | 2 (18%) |  |
|  | 13-15 | 16 (44%) | 7 (25%) | 1 (9%) |  |
|  | Missing | 1 (3%) | 0 | 0 |  |
| GCS motor scale | |  |  |  |  |
|  | 1 | 7 (19%) | 8 (29%) | 5 (45%) | 0.152 |
|  | 2 | 5 (14%) | 4 (14%) | 1 (9%) |  |
|  | 3 | 1 (3%) | 0 | 0 |  |
|  | 4 | 5 (14%) | 1 (4%) | 2 (18%) |  |
|  | 5 | 1 (3%) | 6 (21%) | 1 (9%) |  |
|  | 6 | 17 (47%) | 9 (32%) | 2 (18%) |  |
|  | Missing | 0 | 0 | 0 |  |
| Pupil responsiveness | |  |  |  |  |
|  | Both | 13 (36%) | 7 (25%) | 7 (64%) | 0.181 |
|  | One | 4 (11%) | 4 (14%) | 0 |  |
|  | None | 17 (47%) | 17 (61%) | 3 (27%) |  |
|  | Missing | 2 (6%) | 0 | 1 (9%) |  |
| Hypotension^a, c^ | | 5 (14%) | 7 (25%) | 5 (46%) | 0.091 |
| Hypoxia^d, e^ | | 6 (17%) | 4 (14%) | 3 (27%) | 0.837 |
| Coagulopathy^f, g^ | | 3 (8%) | 2 (7%) | 3 (27%) | 0.128 |
| **Radiology** | |  |  |  |  |
| Perforating | | 11 (31%) | 9 (32%) | 6 (55%) | 0.323 |
| Entry | |  |  |  |  |
|  | Frontobasal | 11 (31%) | 9 (32%) | 6 (55%) | 0.373 |
|  | Temporal | 18 (50%) | 12 (43%) | 5 (46%) |  |
|  | Other | 7 (19%) | 7 (25%) | 0 |  |
| Exit | |  |  |  |  |
|  | Frontobasal | 3 (8%) | 1 (4%) | 3 (27%) | 0.223 |
|  | Temporal | 6 (17%) | 3 (11%) | 2 (18%) |  |
|  | Other | 2 (6%) | 5 (18%) | 1 (9%) |  |
| Trajectory | |  |  |  |  |
|  | Monohemispheric | 19 (53%) | 16 (57%) | 4 (36%) | 0.543 |
|  | Bihemispheric | 16 (44%) | 11 (39%) | 7 (64%) | 0.379 |
|  | Unilobar | 9 (25%) | 6 (21%) | 3 (27%) | 0.874 |
|  | Multilobar | 26 (72%) | 21 (75%) | 8 (73%) | 1.000 |
|  | Posterior fossa | 9 (25%) | 3 (11%) | 2 (18%) | 0.391 |
|  | Transventricular | 14 (39%) | 13 (46%) | 6 (55%) | 0.628 |
|  | In proximity to COW^h^ | 12 (33%) | 9 (32%) | 4 (36%) | 1.000 |
| Bone or projectile fragments present | | 30 (83%) | 24 (86%) | 11 (100%) | 0.525 |
| Basal cisterns | |  |  |  |  |
|  | Normal | 15 (42%) | 9 (32%) | 1 (9%) | 0.140 |
|  | Compressed | 13 (36%) | 16 (57%) | 7 (64%) |  |
|  | Obliterated | 8 (22%) | 3 (11%) | 3 (27%) |  |
| Midline shift | |  |  |  |  |
|  | 0 mm | 22 (61%) | 12 (43%) | 6 (55%) | 0.019 |
|  | 1-5 mm | 3 (8%) | 6 (21%) | 1 (9%) |  |
|  | 5-10 mm | 11 (31%) | 5 (18%) | 1 (9%) |  |
|  | >10 mm | 0 | 5 (18%) | 3 (27%) |  |
| Mass lesion >25 cm^3^ | | 8 (22%) | 8 (29%) | 7 (64%) | 0.046 |
| EDH | | 1 (3%) | 1 (4%) | 0 | 1.000 |
| SDH | | 22 (61%) | 18 (64%) | 8 (73%) | 0.850 |
| ICH | | 26 (72%) | 21 (75%) | 9 (82%) | 0.936 |
| Bilateral SDH | | 4 (11%) | 2 (7%) | 5 (46%) | 0.016 |
| tSAH in convexities | |  |  |  |  |
|  | 0 mm | 7 (19%) | 5 (18%) | 1 (9%) | 0.457 |
|  | 1-5 mm | 10 (28%) | 3 (11%) | 2 (18%) |  |
|  | >5 mm | 19 (53%) | 20 (71%) | 8 (73%) |  |
| tSAH in basal cisterns | |  |  |  |  |
|  | 0 mm | 21 (58%) | 16 (57%) | 4 (36%) | 0.516 |
|  | 1-5 mm | 3 (8%) | 3 (11%) | 3 (27%) |  |
|  | >5 mm | 12 (33%) | 9 (32%) | 4 (36%) |  |
| IVH | | 16 (44%) | 14 (50%) | 9 (82%) | 0.093 |
| Leroux IVH score | |  |  |  |  |
|  | 0 | 20 (56%) | 14 (50%) | 2 (18%) | 0.208 |
|  | 1-10 | 10 (28%) | 7 (25%) | 6 (55%) |  |
|  | >10 | 6 (17%) | 7 (25%) | 3 (27%) |  |
| Acute hydrocephalus | | 5 (14%) | 8 (29%) | 6 (55%) | 0.021 |
| DAI | | 0 | 0 | 0 | NA |
| CTA performed | | 9 (25%) | 7 (25%) | 3 (27%) | 1.000 |
| DSA performed | | 6 (17%) | 3 (11%) | 1 (9%) | 0.898 |
| Confirmed arterial injury | | 4 (11%) | 2 (7%) | 0 | 0.624 |
| Marshall CT classification | |  |  |  |  |
|  | I | 0 | 0 | 0 | 0.168 |
|  | II | 13 (36%) | 8 (29%) | 1 (9%) |  |
|  | III | 8 (22%) | 9 (32%) | 3 (27%) |  |
|  | IV | 7 (19%) | 3 (11%) | 0 |  |
|  | V or VI | 8 (22%) | 8 (29%) | 7 (64%) |  |
| Rotterdam CT score | |  |  |  |  |
|  | 1 | 0 | 0 | 0 | 0.602 |
|  | 2 | 6 (17%) | 3 (11%) | 0 |  |
|  | 3 | 7 (19%) | 5 (18%) | 1 (9%) |  |
|  | 4 | 8 (22%) | 11 (39%) | 4 (36%) |  |
|  | 5 | 13 (36%) | 6 (21%) | 5 (46%) |  |
|  | 6 | 2 (6%) | 3 (11%) | 1 (9%) |  |
| Helsinki CT score | | 5.0 (3.0-8.0) | 5.0 (3.0-8.8) | 10.0 (5.0-12.0) | 0.104 |
| Stockholm CT score | | 3.0 (1.6-4.0) | 3.2 (2.1-4.3) | 4.0 (3.0-5.0) | 0.050 |
| Categorical data presented as N (%) and continuous variables presented as median (IRQ). *Abbreviations*: COW, Circle of Willis; CT, Computerized tomography; CTA, Computerized Tomography Angiography; DAI, Diffuse Axonal Injury; DSA, Digital Subtraction Angiography; EDH, Epidural Hematoma; GCS, Glasgow Coma Scale; ICH, Intracerebral Hematoma; IVH, Intraventricular Hemorrhage; SDH, Subdural Hematoma; tSAH, Traumatic Subarachnoid Hemorrhage  Data missing for ^a^=2, ^b^=1, ^d^=8, ^f^=4 patients  ^c^Systolic blood pressure <90 mmHg at any time prior to admission  ^e^Blood oxygen saturation <90 % at any time prior to admission  ^g^International Normalized Ratio ≥1.5 or Activated Partial Thromboplastin Time >36 s or Thrombocyte Count <100,000 mm^3^  ^h^Within two centimeters of COW | | | | | |
