# Supplementary material for: Prognostic performance of computerized tomography scoring systems in civilian penetrating traumatic brain injury: an observational study
Source: Acta Neurochir (Wien). 2019 Oct 28;161(12):2467–78. doi: 10.1007/s00701-019-04074-1 (PMC6874621; doi:10.1007/s00701-019-04074-1)
Supplement: Supplementary file 5 — Patient baseline characteristics by self-infliction (DOCX 34 kb) [file 701_2019_4074_MOESM5_ESM.docx]

| Parameter | | Self-inflicted injury  (N=48) | Non-self-inflicted injury (N=25) | *p* value |
| --- | --- | --- | --- | --- |
| **Demography** | |  |  |  |
| Age | | 46.5 (32.5-57.8) | 26.0 (21.5-41.5) | <0.001 |
| Sex | |  |  |  |
|  | Male | 47 (98%) | 20 (80%) | 0.016 |
|  | Female | 1 (2%) | 5 (20%) |  |
| **Admission** | |  |  |  |
| Weapon type | |  |  |  |
|  | Firearm | 38 (79%) | 12 (48%) | <0.001 |
|  | Nail gun | 8 (17%) | 2 (8%) |  |
|  | Sharp object | 2 (4%) | 7 (28%) |  |
|  | Other | 0 | 4 (16%) |  |
| Pre-hospital physician involvement^a^ | | 30 (63%) | 19 (79%) | 0.187 |
| Inter-hospital transfer | | 11 (23%) | 2 (8%) | 0.196 |
| Admission delay | |  |  |  |
|  | <1 hour | 12 (25%) | 6 (24%) | 0.559 |
|  | 1-2 hours | 20 (42%9 | 14 (56%) |  |
|  | >2 hours | 14 (29%) | 5 (20%) |  |
|  | Missing | 2 (4%) | 0 |  |
| GCS score | |  |  |  |
|  | 3-8 | 28 (58%) | 11 (44%) | 0.301 |
|  | 9-12 | 7 (15%) | 2 (8%) |  |
|  | 13-15 | 13 (27%) | 11 (44%) |  |
|  | Missing | 0 | 1 (4%) |  |
| GCS motor scale | |  |  |  |
|  | 1 | 14 (29%) | 6 (24%) | 0.418 |
|  | 2 | 6 (13%) | 3 (12%) |  |
|  | 3 | 0 | 1 (4%) |  |
|  | 4 | 6 (13%) | 2 (8%) |  |
|  | 5 | 7 (15%) | 1 (4%) |  |
|  | 6 | 15 (31%) | 12 (48%) |  |
|  | Missing | 0 | 0 |  |
| Pupil responsiveness | |  |  |  |
|  | Both | 23 (48%) | 13 (52%) | 0.095 |
|  | One | 8 (17%) | 0 |  |
|  | None | 16 (33%) | 10 (40%) |  |
|  | Missing | 1 (2%) | 2 (8%) |  |
| Hypotension^b, c^ | | 11 (23%) | 5 (20%) | 0.775 |
| Hypoxia^d, e^ | | 7 (15%) | 5 (20%) | 0.515 |
| Coagulopathy^f, g^ | | 5 (10%) | 2 (8%) | 1.000 |
| **Radiology** | |  |  |  |
| Perforating | | 19 (40%) | 7 (28%) | 0.441 |
| Entry | |  |  |  |
|  | Frontobasal | 21 (44%) | 4 (16%) | 0.025 |
|  | Temporal | 21 (44%) | 13 (52%) |  |
|  | Other | 6 (13%) | 8 (32%) |  |
| Exit | |  |  |  |
|  | Frontobasal | 5 (10%) | 2 (8%) | 0.866 |
|  | Temporal | 8 (17%) | 3 (12%) |  |
|  | Other | 6 (13%) | 2 (8%) |  |
| Trajectory | |  |  |  |
|  | Monohemispheric | 21 (44%) | 18 (72%) | 0.027 |
|  | Bihemispheric | 26 (54%) | 6 (24%) | 0.024 |
|  | Unilobar | 10 (21%) | 8 (32%) | 0.392 |
|  | Multilobar | 37 (77%) | 16 (64%) | 0.275 |
|  | Posterior fossa | 6 (13%) | 6 (24%) | 0.318 |
|  | Transventricular | 26 (54%) | 6 (24%) | 0.024 |
|  | In proximity to COW^h^ | 20 (42%) | 5 (20%) | 0.075 |
| Bone or projectile fragments present | | 46 (96%) | 18 (72%) | 0.006 |
| Basal cisterns | |  |  |  |
|  | Normal | 12 (25%) | 13 (52%) | 0.046 |
|  | Compressed | 27 (56%) | 7 (28%) |  |
|  | Obliterated | 9 (19%) | 5 (20%) |  |
| Midline shift | |  |  |  |
|  | 0 mm | 25 (52%) | 15 (60%) | 0.330 |
|  | 1-5 mm | 8 (17%) | 2 (8%) |  |
|  | 5-10 mm | 8 (17%) | 7 (28%) |  |
|  | >10 mm | 7 (15%) | 1 (4%) |  |
| Mass lesion >25 cm^3^ | | 15 (31%) | 8 (32%) | 1.000 |
| EDH | | 0 | 2 (8%) | 0.114 |
| SDH | | 33 (69%) | 13 (52%) | 0.204 |
| ICH | | 39 (81%) | 16 (64%) | 0.152 |
| Bilateral SDH | | 8 (17%) | 3 (12%) | 0.738 |
| tSAH in convexities | |  |  |  |
|  | 0 mm | 9 (19%) | 4 (16%) | 0.002 |
|  | 1-5 mm | 3 (6%) | 10 (40%) |  |
|  | >5 mm | 36 (75%) | 11 (44%) |  |
| tSAH in basal cisterns | |  |  |  |
|  | 0 mm | 22 (46%) | 18 (72%) | 0.117 |
|  | 1-5 mm | 7 (15%) | 2 (8%) |  |
|  | >5 mm | 19 (40%) | 5 (20%) |  |
| IVH | | 28 (58%) | 9 (36%) | 0.087 |
| Leroux IVH score | |  |  |  |
|  | 0 | 20 (42%) | 16 (64%) | 0.166 |
|  | 1-10 | 15 (31%) | 6 (24%) |  |
|  | >10 | 13 (27%) | 3 (12%) |  |
| Acute hydrocephalus | | 13 (27%) | 4 (16%) | 0.386 |
| DAI | | 0 | 0 | NA |
| CTA performed | | 13 (27%) | 6 (24%) | 1.000 |
| DSA performed | | 4 (15%) | 3 (12%) | 1.000 |
| Confirmed arterial injury | | 3 (6%) | 3 (12%) | 0.406 |
| Marshall CT classification | |  |  |  |
|  | I | 0 | 0 | 0.114 |
|  | II | 11 (23%) | 11 (44%) |  |
|  | III | 17 (35%) | 3 (12%) |  |
|  | IV | 5 (10%) | 3 (12%) |  |
|  | V or VI | 15 (31%) | 8 (32%) |  |
| Rotterdam CT score | |  |  |  |
|  | 1 | 0 | 0 | 0.358 |
|  | 2 | 4 (8%) | 5 (20%) |  |
|  | 3 | 7 (15%) | 6 (24%) |  |
|  | 4 | 18 (38%) | 5 (20%) |  |
|  | 5 | 15 (31%) | 7 (28%) |  |
|  | 6 | 4 (8%) | 2 (8%) |  |
| Helsinki CT score | | 7.0 (4.0-10.0) | 4.0 (1.0-8.5) | 0.110 |
| Stockholm CT score | | 3.5 (2.5-4.3) | 2.3 (1.5-4.0) | 0.022 |
| Categorical data presented as N (%) and continuous variables presented as median (IRQ). *Abbreviations*: COW, Circle of Willis; CT, Computerized tomography; CTA, Computerized Tomography Angiography; DAI, Diffuse Axonal Injury; DSA, Digital Subtraction Angiography; EDH, Epidural Hematoma; GCS, Glasgow Coma Scale; ICH, Intracerebral Hematoma; IVH, Intraventricular Hemorrhage; SDH, Subdural Hematoma; tSAH, Traumatic Subarachnoid Hemorrhage  Data missing for a=^1^, ^b^=2, ^d^=7, ^f^=4 patients  ^c^Systolic blood pressure <90 mmHg at any time prior to admission  ^e^Blood oxygen saturation <90 % at any time prior to admission  ^g^International Normalized Ratio ≥1.5 or Activated Partial Thromboplastin Time >36 s or Thrombocyte Count <100,000 mm^3^  ^h^Within two centimeters of COW | | | | |
