# Supplementary material for: Prognostic performance of computerized tomography scoring systems in civilian penetrating traumatic brain injury: an observational study
Source: Acta Neurochir (Wien). 2019 Oct 28;161(12):2467–78. doi: 10.1007/s00701-019-04074-1 (PMC6874621; doi:10.1007/s00701-019-04074-1)
Supplement: Supplementary file 6 — Treatment characteristics (DOCX 23 kb) [file 701_2019_4074_MOESM6_ESM.docx]

| Parameter | Active treatment cohort  (N=59) |
| --- | --- |
| **Treatment** |  |
| Mechanical ventilation | 54 (92%) |
| Tracheostomy^f^ | 16 (27%) |
| ICP-monitoring | 25 (42%) |
| External ventricular drainage | 13 (22%) |
| Debridement | 45 (76%) |
| Craniotomy and hematoma evacuation | 16 (27%) |
| Decompressive craniectomy | 4 (7%) |
| Fracture elevation or reconstruction | 11 (19%) |
| Other operation | 14 (24%) |
| ICU length of stay (days) | 4.7 (1.0-10.0) |
| Hospital length of stay (days) | 8.0 (5.0-17.0) |
| Categorical data presented as N (%) and continuous variables presented as median (IRQ). Abbreviations: ICP; Intracranial Pressure; ICU, Intensive Care Unit  ^f^Data missing for 4 patients | |
