# Supplementary material for: Prognostic performance of computerized tomography scoring systems in civilian penetrating traumatic brain injury: an observational study
Source: Acta Neurochir (Wien). 2019 Oct 28;161(12):2467–78. doi: 10.1007/s00701-019-04074-1 (PMC6874621; doi:10.1007/s00701-019-04074-1)
Supplement: Supplementary file 7 — Patient baseline characteristics by GCS (DOCX 35 kb) [file 701_2019_4074_MOESM7_ESM.docx]

| Parameter | | GCS 3-8  (N=40) | GCS 9-12  (N=10) | GCS 13-15  (N=24) | *p* value |
| --- | --- | --- | --- | --- | --- |
| **Demography** | |  |  |  |  |
| Age | | 41.5 (28.0-55.8) | 49.0 (40.5-60.5) | 33.5 (22.3-47.8) | 0.052 |
| Sex | |  |  |  |  |
|  | Male | 37 (93%) | 8 (80%) | 22 (92%) | 0.457 |
|  | Female | 3 (8%) | 2 (20%) | 2 (8%) |  |
| **Admission** | |  |  |  |  |
| Weapon type | |  |  |  |  |
|  | Firearm | 35 (88%) | 6 (60%) | 10 (42%) | 0.001 |
|  | Nail gun | 3 (8%) | 0 | 7 (29%) |  |
|  | Sharp object | 2 (5%) | 3 (30%) | 4 (17%) |  |
|  | Other | 0 | 1 (10%) | 3 (13%) |  |
| Self-inflicted injury^a^ | | 28 (72%) | 7 (78%) | 13 (54%) | 0.301 |
| Pre-hospital physician involvement^b^ | | 33 (83%) | 4 (40%) | 13 (57%) | 0.010 |
| Inter-hospital transfer | | 3 (8%) | 3 (30%) | 8 (33%) | 0.015 |
| Admission delay | |  |  |  |  |
|  | <1 hour | 8 (21%) | 3 (30%) | 7 (30%) | 0.178 |
|  | 1-2 hours | 24 (62%) | 3 (30%) | 8 (35%) |  |
|  | >2 hours | 7 (18%) | 4 (40%) | 8 (35%) |  |
|  | Missing | 1 (3%) | 0 | 1 (4%) |  |
| Pupil responsiveness | |  |  |  |  |
|  | Both | 15 (39%) | 4 (40%) | 17 (77%) | 0.013 |
|  | One | 4 (10%) | 3 (30%) | 1 (4%) |  |
|  | None | 20 (51%) | 3 (30%) | 4 (18%) |  |
|  | Missing | 1 (3%) | 0 | 2 (8%) |  |
| Hypotension^a, c^ | | 13 (33%) | 2 (20%) | 2 (8%) | 0.052 |
| Hypoxia^d, e^ | | 11 (28%) | 1 (10%) | 1 (4%) | 0.023 |
| Coagulopathy^f, g^ | | 5 (13%) | 2 (20%) | 1 (4%) |  |
| **Radiology** | |  |  |  |  |
| Perforating | | 22 (55%) | 2 (20%) | 2 (8%) | <0.001 |
| Entry | |  |  |  |  |
|  | Frontobasal | 17 (43%) | 5 (50%) | 4 (17%) | 0.018 |
|  | Temporal | 20 (50%) | 3 (30%) | 11 (46%) |  |
|  | Other | 3 (8%) | 2 (20%) | 9 (38%) |  |
| Exit | |  |  |  |  |
|  | Frontobasal | 5 (13%) | 1 (10%) | 1 (4%) | 0.003 |
|  | Temporal | 11 (28%) | 0 | 0 |  |
|  | Other | 6 (15%) | 1 (10%) | 1 (4%) |  |
| Trajectory | |  |  |  |  |
|  | Monohemispheric | 13 (33%) | 7 (70%) | 18 (75%) | 0.002 |
|  | Bihemispheric | 26 (65%) | 3 (30%) | 5 (21%) | 0.001 |
|  | Unilobar | 4 (10%) | 2 (20%) | 12 (50%) | 0.001 |
|  | Multilobar | 35 (88%) | 8 (80%) | 11 (46%) | 0.001 |
|  | Posterior fossa | 10 (25%) | 2 (20%) | 2 (8%) | 0.294 |
|  | Transventricular | 24 (60%) | 3 (30%) | 6 (25%) | 0.015 |
|  | In proximity to COW^h^ | 15 (38%) | 3 (30%) | 7 (29%) | 0.787 |
| Bone or projectile fragments present | | 39 (98%) | 7 (70%) | 19 (79%) | 0.007 |
| Basal cisterns | |  |  |  |  |
|  | Normal | 4 (10%) | 2 (20%) | 19 (79%) | <0.001 |
|  | Compressed | 22 (55%) | 8 (80%) | 5 (21%) |  |
|  | Obliterated | 14 (35%) | 0 | 0 |  |
| Midline shift | |  |  |  |  |
|  | 0 mm | 20 (50%) | 3 (30%) | 17 (71%) | 0.006 |
|  | 1-5 mm | 3 (8%) | 2 (20%) | 5 (21%) |  |
|  | 5-10 mm | 9 (23%) | 5 (50%) | 2 (8%) |  |
|  | >10 mm | 8 (20%) | 0 | 0 |  |
| Mass lesion >25 cm^3^ | | 20 (50%) | 2 (20%) | 0 | <0.001 |
| EDH | | 0 | 1 (10%) | 1 (4%) | 0.106 |
| SDH | | 31 (78%) | 7 (70%) | 10 (42%) | 0.017 |
| ICH | | 33 (83%) | 8 (80%) | 14 (58%) | 0.106 |
| Bilateral SDH | | 10 (25%) | 1 (10%) | 0 | 0.015 |
| tSAH in convexities | |  |  |  |  |
|  | 0 mm | 5 (13%) | 1 (10%) | 7 (29%) | 0.204 |
|  | 1-5 mm | 6 (15%) | 3 (30%) | 6 (25%) |  |
|  | >5 mm | 29 (73%) | 6 (60%) | 11 (46%) |  |
| tSAH in basal cisterns | |  |  |  |  |
|  | 0 mm | 15 (38%) | 4 (40%) | 22 (92%) | <0.001 |
|  | 1-5 mm | 7 (18%) | 1 (10%) | 1 (4%) |  |
|  | >5 mm | 18 (45%) | 5 (50%) | 1 (4%) |  |
| IVH | | 31 (78%) | 4 (40%) | 4 (17%) | <0.001 |
| Leroux IVH score | |  |  |  |  |
|  | 0 | 9 (23%) | 6 (60%) | 20 (83%) | <0.001 |
|  | 1-10 | 17 (43%) | 2 (20%) | 4 (17%) |  |
|  | >10 | 14 (35%) | 2 (20%) | 0 |  |
| Acute hydrocephalus | | 14 (35%) | 4 (40%) | 1 (4%) | 0.007 |
| DAI | | 0 | 0 | 0 | NA |
| CTA performed | | 9 (23%) | 4 (40%) | 6 (25%) | 0.571 |
| DSA performed | | 8 (20%) | 1 (10%) | 1 (4%) | 0.165 |
| Confirmed arterial injury | | 5 (13%) | 0 | 1 (4%) | 0.490 |
| Marshall CT classification | |  |  |  |  |
|  | I | 0 | 0 | 0 | <0.001 |
|  | II | 3 (8%) | 1 (10%) | 18 (75%) |  |
|  | III | 13 (33%) | 3 (30%) | 4 (17%) |  |
|  | IV | 4 (10%) | 4 (40%) | 2 (8%) |  |
|  | V or VI | 20 (50%) | 2 (20%) | 0 |  |
| Rotterdam CT score | |  |  |  |  |
|  | 1 | 0 | 0 | 0 | <0.001 |
|  | 2 | 1 (3%) | 1 (10%) | 7 (29%) |  |
|  | 3 | 2 (5%) | 0 | 11 (46%) |  |
|  | 4 | 13 (33%) | 5 (50%) | 5 (21%) |  |
|  | 5 | 18 (45%) | 4 (40%) | 1 (4%) |  |
|  | 6 | 6 (15%) | 0 | 0 |  |
| Helsinki CT score | | 8.5 (7.0-12.0) | 5.0 (2.5-8.3) | 3.0 (0.0-4.0) | <0.001 |
| Stockholm CT score | | 4.0 (3.0-4.7) | 3.3 (2.0-4.6) | 2.0 (1.0-2.5) | <0.001 |
| Categorical data presented as N (%) and continuous variables presented as median (IRQ). *Abbreviations*: COW, Circle of Willis; CT, Computerized tomography; CTA, Computerized Tomography Angiography; DAI, Diffuse Axonal Injury; DSA, Digital Subtraction Angiography; EDH, Epidural Hematoma; GCS, Glasgow Coma Scale; ICH, Intracerebral Hematoma; IVH, Intraventricular Hemorrhage; SDH, Subdural Hematoma; tSAH, Traumatic Subarachnoid Hemorrhage  Data missing for ^a^=2, ^b^=1, ^d^=8, ^f^=4 patients  ^c^Systolic blood pressure <90 mmHg at any time prior to admission  ^e^Blood oxygen saturation <90 % at any time prior to admission  ^g^International Normalized Ratio ≥1.5 or Activated Partial Thromboplastin Time >36 s or Thrombocyte Count <100,000 mm^3^  ^h^Within two centimeters of COW | | | | | |
