# Supplementary material for: Prognostic performance of computerized tomography scoring systems in civilian penetrating traumatic brain injury: an observational study
Source: Acta Neurochir (Wien). 2019 Oct 28;161(12):2467–78. doi: 10.1007/s00701-019-04074-1 (PMC6874621; doi:10.1007/s00701-019-04074-1)
Supplement: Supplementary file 8 — Patient baseline characteristics by weapon (DOCX 38 kb) [file 701_2019_4074_MOESM8_ESM.docx]

| Parameter | | Firearm (N=51) | Nail gun (N=10) | Sharp object (N=10) | Other  (N=4) | *p* value |
| --- | --- | --- | --- | --- | --- | --- |
| **Demography** | |  |  |  |  |  |
| Age | | 44.0 (28.0-56.0) | 44.0 (25.0-53.5) | 24.0 (20.8-39.5) | 31.0 (16.3-47.3) | 0.024 |
| Sex | |  |  |  |  |  |
|  | Male | 48 (94%) | 10 (100%) | 7 (70%) | 3 (75%) | 0.047 |
|  | Female | 3 (6%) | 0 | 3 (30%) | 1 (25%) |  |
| **Admission** | |  |  |  |  |  |
| Self-inflicted injury^a^ | | 38 (75%) | 8 (80%) | 2 (20%) | 0 | <0.001 |
| Pre-hospital physician involvement^b^ | | 34 (67%) | 5 (50%) | 8 (80%) | 4 (100%) | 0.318 |
| Inter-hospital transfer | | 10 (20%) | 2 (20%) | 2 (20%) | 0 | 1.000 |
| Admission delay | |  |  |  |  |  |
|  | <1 hour | 12 (24%) | 3 (30%) | 2 (20%) | 1 (25%) | 0.937 |
|  | 1-2 hours | 24 (47%) | 5 (50%) | 6 (60%) | 1 (25%) |  |
|  | >2 hours | 13 (25%) | 2 (20%) | 2 (20%) | 2 (50%) |  |
|  | Missing | 2 (4%) | 0 | 0 | 0 |  |
| GCS score | |  |  |  |  |  |
|  | 3-8 | 35 (69%) | 3 (30%) | 2 (20%) | 0 | 0.001 |
|  | 9-12 | 6 (12%) | 0 | 3 (30%) | 1 (25%) |  |
|  | 13-15 | 10 (20%) | 7 (70%) | 4 (40%) | 3 (75%) |  |
|  | Missing | 0 | 0 | 1 (10%) | 0 |  |
| GCS motor scale | |  |  |  |  |  |
|  | 1 | 19 (37%) | 0 | 1 (10%) | 0 | 0.089 |
|  | 2 | 8 (16%) | 1 (10%) | 1 (10%) | 0 |  |
|  | 3 | 1 (2%) | 0 | 0 | 0 |  |
|  | 4 | 6 (12%) | 1 (10%) | 1 (10%) | 0 |  |
|  | 5 | 5 (10%) | 1 (10%) | 2 (20%) | 0 |  |
|  | 6 | 12 (24%) | 7 (70%) | 5 (50%) | 4 (100%) |  |
|  | Missing | 0 | 0 | 0 | 0 |  |
| Pupil responsiveness | |  |  |  |  |  |
|  | Both | 20 (39%) | 10 (100%) | 5 (50%) | 2 (50%) | 0.031 |
|  | One | 7 (14%) | 0 | 1 (10%) | 0 |  |
|  | None | 22 (43%) | 0 | 3 (30%) | 2 (50%) |  |
|  | Missing | 2 (4%) | 0 | 1 (10%) | 0 |  |
| Hypotension^a, c^ | | 14 (27%) | 0 | 3 (30%) | 0 | 0.215 |
| Hypoxia^d, e^ | | 11 (22%) | 1 (10%) | 0 | 1 (25%) | 0.380 |
| Coagulopathy^f, g^ | | 6 (12%) | 0 | 1 (10%) | 1 (25%) | 0.377 |
| **Radiology** | |  |  |  |  |  |
| Perforating | | 24 (47%) | 2 (20%) | 0 | 0 | 0.004 |
| Entry | |  |  |  |  |  |
|  | Frontobasal | 22 (43%) | 0 | 4 (40%) | 0 | 0.016 |
|  | Temporal | 23 (45%) | 5 (50%) | 4 (40%) | 3 (75%) |  |
|  | Other | 6 (12%) | 5 (50%) | 2 (20%) | 1 (25%) |  |
| Exit | |  |  |  |  |  |
|  | Frontobasal | 7 (14%) | 0 | 0 | 0 | 0.260 |
|  | Temporal | 9 (18%) | 2 (20%) | 0 | 0 |  |
|  | Other | 8 (16%) | 0 | 0 | 0 |  |
| Trajectory | |  |  |  |  |  |
|  | Monohemispheric | 21 (41%) | 6 (60%) | 8 (80%) | 4 (100%) | 0.017 |
|  | Bihemispheric | 28 (55%) | 4 (40%) | 2 (20%) | 0 | 0.047 |
|  | Unilobar | 7 (14%) | 5 (50%) | 4 (40%) | 2 (20%) | 0.012 |
|  | Multilobar | 42 (82%) | 5 (50%) | 6 (60%) | 2 (50%) | 0.043 |
|  | Posterior fossa | 11 (22%) | 1 (10%) | 2 (20%) | 0 | 0.828 |
|  | Transventricular | 24 (47%) | 5 (50%) | 4 (40%) | 0 | 0.384 |
|  | In proximity to COW^h^ | 16 (31%) | 4 (40%) | 5 (50%) | 0 | 0.371 |
| Bone or projectile fragments present | | 50 (98%) | 10 (100%) | 3 (30%) | 2 (50%) | <0.001 |
| Basal cisterns | |  |  |  |  |  |
|  | Normal | 10 (20%) | 6 (60%) | 5 (50%) | 4 (100%) | 0.006 |
|  | Compressed | 28 (55%) | 4 (40%) | 4 (40%) | 0 |  |
|  | Obliterated | 13 (26%) | 0 | 1 (10%) | 0 |  |
| Midline shift | |  |  |  |  |  |
|  | 0 mm | 25 (49%) | 9 (90%) | 3 (30%) | 3 (75%) | 0.291 |
|  | 1-5 mm | 7 (14%) | 1 (10%) | 2 (20%) | 0 |  |
|  | 5-10 mm | 12 (24%) | 0 | 4 (40%) | 1 (25%) |  |
|  | >10 mm | 7 (14%) | 0 | 1 (10%) | 0 |  |
| Mass lesion >25 cm^3^ | | 20 (39%) | 0 | 3 (30%) | 0 | 0.038 |
| EDH | | 1 (2%) | 0 | 0 | 1 (25%) | 0.173 |
| SDH | | 41 (80%) | 1 (10%) | 5 (50%) | 1 (25%) | <0.001 |
| ICH | | 45 (88%) | 2 (20%) | 7 (70%) | 2 (50%) | <0.001 |
| Bilateral SDH | | 11 (22%) | 0 | 0 | 0 | 0.151 |
| tSAH in convexities | |  |  |  |  |  |
|  | 0 mm | 6 (12%) | 4 (40%) | 3 (30%) | 0 | 0.060 |
|  | 1-5 mm | 10 (20%) | 0 | 3 (30%) | 2 (50%) |  |
|  | >5 mm | 35 (69%) | 6 (60%) | 4 (40%) | 2 (50%) |  |
| tSAH in basal cisterns | |  |  |  |  |  |
|  | 0 mm | 25 (49%) | 7 (70%) | 5 (50%) | 4 (100%) | 0.307 |
|  | 1-5 mm | 9 (18%) | 0 | 0 | 0 |  |
|  | >5 mm | 17 (33%) | 3 (30%) | 5 (50%) | 0 |  |
| IVH | | 31 (61%) | 3 (30%) | 5 (50%) | 0 | 0.052 |
| Leroux IVH score | |  |  |  |  |  |
|  | 0 | 20 (39%) | 7 (70%) | 5 (50%) | 4 (100%) | 0.270 |
|  | 1-10 | 17 (33%) | 2 (20%) | 4 (40%) | 0 |  |
|  | >10 | 14 (28%) | 1 (10%) | 1 (10%) | 0 |  |
| Acute hydrocephalus | | 15 (29%) | 1 (10%) | 3 (30%) | 0 | 0.449 |
| DAI | | 0 | 0 | 0 | 0 | NA |
| CTA performed | | 10 (20%) | 3 (30%) | 4 (40%) | 2 (50%) | 0.259 |
| DSA performed | | 5 (10%) | 2 (20%) | 3 (30%) | 0 | 0.241 |
| Confirmed arterial injury | | 4 (8%) | 0 | 1 (10%) | 1 (25%) | 0.441 |
| Marshall CT classification | |  |  |  |  |  |
|  | I | 0 | 0 | 0 | 0 | 0.007 |
|  | II | 9 (18%) | 6 (60%) | 4 (40%) | 3 (75%) |  |
|  | III | 15 (29%) | 4 (40%) | 1 (10%) | 0 |  |
|  | IV | 7 (14%) | 0 | 2 (20%) | 1 (25%) |  |
|  | V or VI | 20 (39%) | 0 | 3 (30%) | 0 |  |
| Rotterdam CT score | |  |  |  |  |  |
|  | 1 | 0 | 0 | 0 | 0 | 0.015 |
|  | 2 | 3 (6%) | 3 (30%) | 2 (20%) | 1 (25%) |  |
|  | 3 | 6 (12%) | 3 (30%) | 2 (20%) | 2 (50%) |  |
|  | 4 | 17 (33%) | 4 (40%) | 1 (10%) | 1 (25%) |  |
|  | 5 | 19 (37%) | 0 | 5 (50%) | 0 |  |
|  | 6 | 6 (12%) | 0 | 0 | 0 |  |
| Helsinki CT score | | 8.0 (4.0-10.0) | 1.5 (0.0-3.3) | 5.0 (3.0-8.3) | 1.0 (-2.3-3.5) | <0.001 |
| Stockholm CT score | | 3.5 (2.5-4.5) | 2.0 (1.0-3.3) | 3.6 (1.8-4.1) | 1.8 (0.8-2.8) | 0.012 |
| Categorical data presented as N (%) and continuous variables presented as median (IRQ). *Abbreviations*: COW, Circle of Willis; CT, Computerized tomography; CTA, Computerized Tomography Angiography; DAI, Diffuse Axonal Injury; DSA, Digital Subtraction Angiography; EDH, Epidural Hematoma; GCS, Glasgow Coma Scale; ICH, Intracerebral Hematoma; IVH, Intraventricular Hemorrhage; SDH, Subdural Hematoma; tSAH, Traumatic Subarachnoid Hemorrhage  Data missing for ^a^=2, ^b^=1, ^d^=8, ^f^=4 patients  ^c^Systolic blood pressure <90 mmHg at any time prior to admission  ^e^Blood oxygen saturation <90 % at any time prior to admission  ^g^International Normalized Ratio ≥1.5 or Activated Partial Thromboplastin Time >36 s or Thrombocyte Count <100,000 mm^3^  ^h^Within two centimeters of COW | | | | | | |
